# Pangenome Analysis of a Salmonella Enteritidis Population Links a Major Outbreak to a Gifsy-1-Like Prophage Containing Anti-Inflammatory Gene *gogB*

**DOI:** 10.1128/spectrum.02791-22

**Published:** 2023-03-14

**Authors:** Adam J. Svahn, Carl J. E. Suster, Sheryl L. Chang, Rebecca J. Rockett, Eby M. Sim, Oliver M. Cliff, Qinning Wang, Alicia Arnott, Marc Ramsperger, Tania C. Sorrell, Vitali Sintchenko, Mikhail Prokopenko

**Affiliations:** a Centre for Complex Systems, Faculty of Engineering, The University of Sydney, Sydney, NSW, Australia; b Sydney Infectious Diseases Institute, The University of Sydney, Westmead, NSW, Australia; c Centre for Infectious Diseases and Microbiology–Public Health, Westmead Hospital, Westmead, New South Wales, Australia; d School of Physics, Faculty of Science, The University of Sydney, Sydney, NSW, Australia; e Institute of Clinical Pathology and Medical Research, NSW Health Pathology, Westmead, NSW, Australia; Institut National de Santé Publique du Québec

**Keywords:** *Salmonella Enteritidis*, foodborne pathogen, pangenome, prophage, virulence factor, accessory genome, whole-genome sequencing, gogB

## Abstract

A major outbreak of the globally significant Salmonella Enteritidis foodborne pathogen was identified within a large clinical data set by a program of routine WGS of clinical presentations of salmonellosis in New South Wales, Australia. Pangenome analysis helped to quantify and isolate prophage content within the accessory partition of the pangenome. A prophage similar to Gifsy-1 (henceforth GF-1L) was found to occur in all isolates of the outbreak core SNP cluster, and in three other isolates. Further analysis revealed that the GF-1L prophage carried the *gogB* virulence factor. These observations suggest that GF-1L may be an important marker of virulence for S. Enteritidis population screening and, that anti-inflammatory, *gogB*-mediated virulence currently associated with Salmonella *Typhimurium* may also be displayed by S. Enteritidis.

**IMPORTANCE** We examined 5 years of genomic and epidemiological data for the significant global foodborne pathogen, Salmonella enterica. Although Salmonella enterica subspecies enterica serovar Enteritidis (S. Enteritidis) is the leading cause of salmonellosis in the USA and Europe, prior to 2018 it was not endemic in the southern states of Australia. However, in 2018 a large outbreak led to the endemicity of S. Enteritidis in New South Wales, Australia, and a unique opportunity to study this phenomenon. Using pangenome analysis we uncovered that this clone contained a Gifsy-1-like prophage harboring the known virulence factor *gogB.* The prophage reported has not previously been described in S. Enteritidis isolates.

## OBSERVATION

Salmonella infections inflict a heavy toll on global public health, affecting 78.5 million people globally each year (1). Two nontyphoidal Salmonella enterica serovars, S. Enteritidis and *S. Typhimurium*, cause the majority of infections (2). The emerging use of whole-genome sequencing (WGS) for public health laboratory surveillance of Salmonella provides the opportunity to examine the genetic content across the entire pangenome. However, clustering of Salmonella genomes for outbreak analysis relies mainly on the core partition of the pangenome (the genome content which is shared by the majority of isolates). A major driver of Salmonella pathogenicity is the acquisition of virulence genes via horizontal transfer, which represents the accessory part of the pangenome and which is often excluded from routine analysis of outbreak strains. Acquisition of virulence genes in S. enterica serovars can be mediated by temperate bacteriophages. Specifically, in *S. Typhimurium* (STM) two lambdoid phages, Gifsy-1 and Gifsy-2, carrying several known virulence genes are commonly acquired (3). Understanding accessory genome dynamics can provide important additional information for risk assessment and public health investigation of Salmonella outbreaks and differentiation between the outbreak and sporadic strains of Salmonella.

We report here an application of pangenome analysis using a community outbreak of foodborne S. Enteritidis in New South Wales (NSW), Australia, between May 2018 and January 2020. During this period all clinical isolates of S. Enteritidis diagnosed in NSW were subjected to routine WGS as part of public health laboratory surveillance (4). Pangenome analysis of the S. Enteritidis population with a focus on the accessory partition of the pangenome highlighted a significant proportion of accessory variance consisted of prophage repertoire and, significantly, a Gifsy-1-like prophage carrying the virulence factor *gogB* found uniquely in isolates associated with the outbreak.

Between 2016 and 2019 1,150 clinical isolates of S. Enteritidis were referred to the NSW Enteric Reference Laboratory, Institute of Clinical Pathology and Medical Research (ICPMR), NSW Health Pathology, for WGS. Sequencing and bioinformatic analysis were conducted as detailed in a network analysis of this population (4), resulting in 897 genomes passing quality requirements. The core genome was defined using Roary (version 3.12.0) with default parameters (5) and core SNP differences between isolates were quantified using SNP-sites (6). Core SNP clusters were defined by considering isolates chronologically: if an isolate was <10 core SNPs distant from the index isolate of an existing cluster it was assigned to that cluster, otherwise it was designated the index isolate of a putative new SNP cluster. To investigate the accessory partition of the pangenome, a dynamic pangenome partitioning and a pangenome graph were generated with PPanGGOLiN (version 1.0.13) (7).

**FIG 1:**
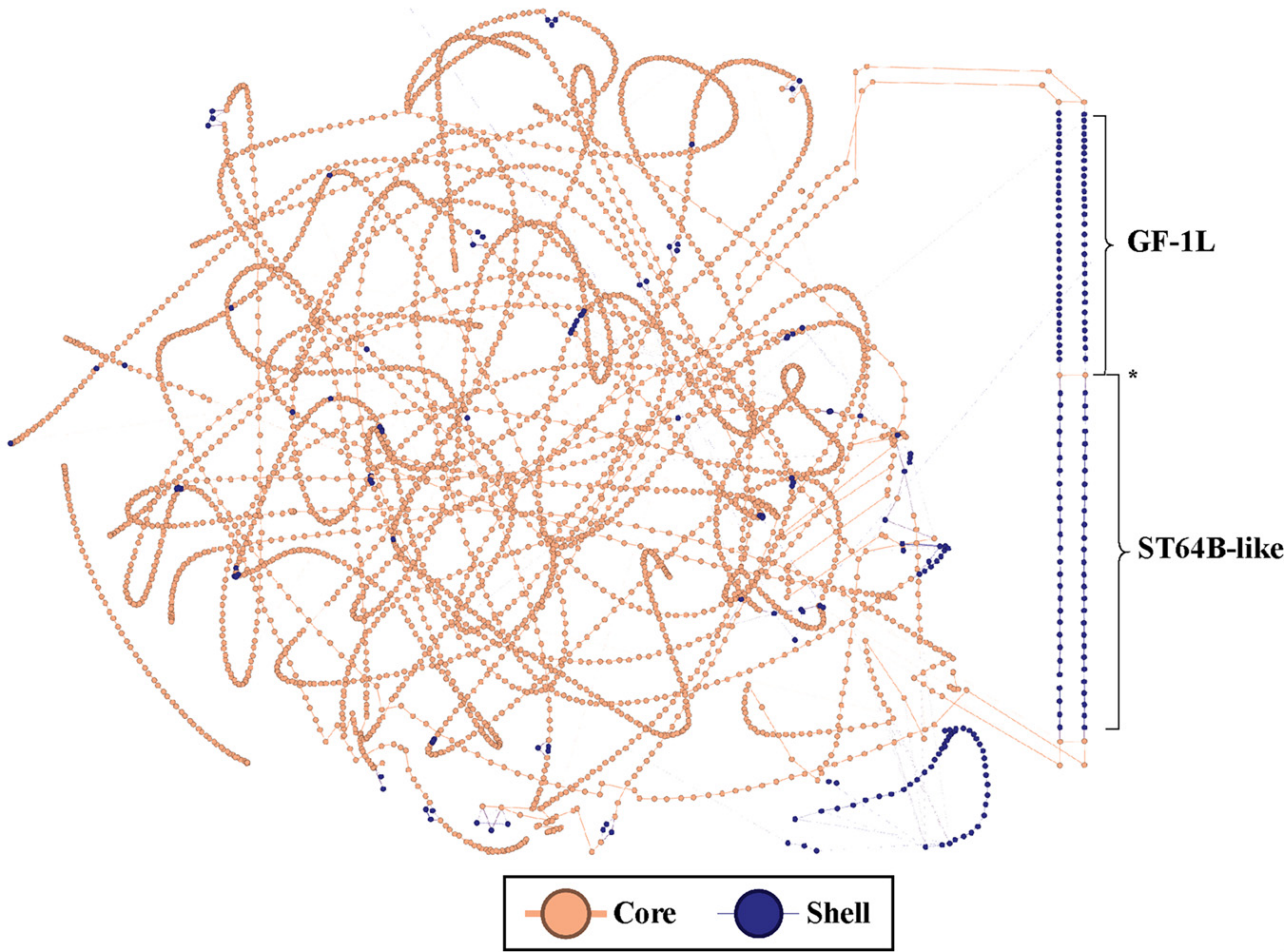
Pangenome graph of the S. Enteritidis population of this study. Each node represents a gene family, and edges connect gene families containing genes that are contiguous on the genomes. Node color indicates the pangenome partition. Cloud accessory gene families are not shown. The network layout is force-based, with the paths corresponding to the two prophages indicated and visually separated. * indicates a pair of genes (putative holins) that are shared by the GF-1L and ST64B-like prophages.

An S. Enteritidis outbreak was declared in NSW in 2018 (8), with detections at 16 farms in NSW and five in the neighboring southern state of Victoria (9, 10). Surveillance of S. Enteritidis at poultry facilities was expanded in response to its transition to endemic status. The outbreak has previously been contextualized within global S. Enteritidis lineages (11). As previously reported (4), the total NSW S. Enteritidis population was found to consist of 78 unique SNP clusters, including the outbreak-associated cluster, SalEnt-18-0030, containing 197 isolates collected from May 2018 onwards. Pangenome analysis of the S. Enteritidis population with PPanGGOLiN identified 11,839 gene families, and its network-based partitioning method classified 4,288 of these gene families as core (present in 87 to 100% of isolates), 278 as shell accessory (present in 11 to 87% of isolates) and 7,273 as cloud accessory (present in 0.001 to 11% of isolates). A pangenome graph was generated in which gene families (high similarity gene alignments) are represented as nodes and genomic colocalization is represented as edges (i.e., genes which are neighbors in at least one genome are linked by an edge). Each individual genome can be considered one of many paths through the pangenome graph. Inspection of this pangenome graph (Fig. 1) showed that two large contiguous regions made up a significant portion of the shell component of the pangenome (118/278 gene families, 43%).

Prophage scanning of these regions using PHASTER (12) predicted an intact 51.4 kb prophage with high nucleotide similarity to Gifsy-1 (NC_010392.1) and a 62.7 kb prophage with the best similarity to 118970_sal3 (NC_031940.1). Manual curation of the second prophage (henceforth referred to as a ST64B-like prophage) from a representative sequence revealed that it was actually a 40.6 kb prophage with BLASTN hits to not only 118970_sal3 (96.82% nucleotide identity over 63% query coverage) but also the temperate phage ST64B (98.60% nucleotide identity over 62% query coverage; Fig. S1). The prophage inserted into the tRNA gene *serU*, previously noted as the insertion site for ST64B (13). The ST64B-like prophage occurred in isolates across multiple SNP clusters but did not occur in any members of SalEnt-18-0030.

**FIG 2:**
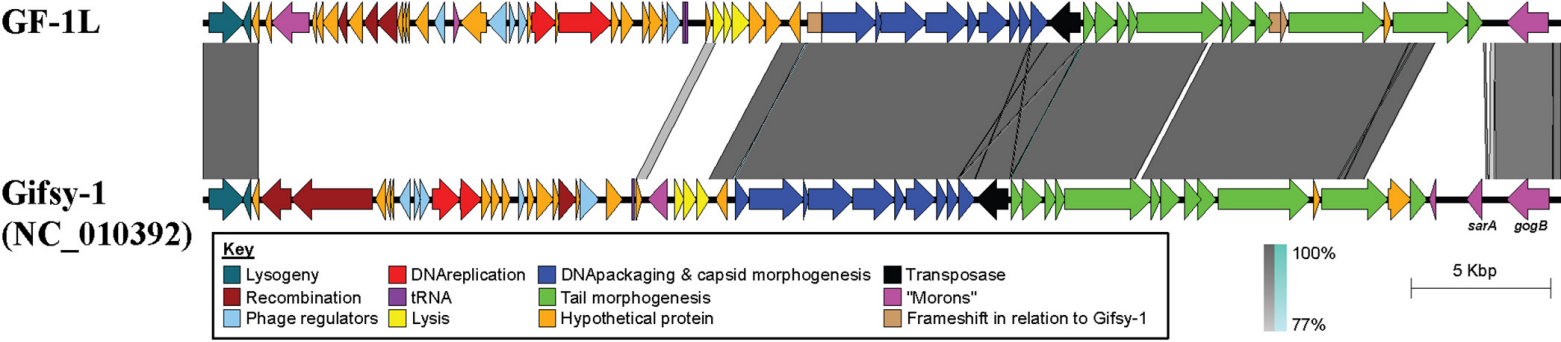
Pairwise comparison between the outbreak associated prophage (Top; most common GF-1L sequence) and reference phage Gifsy-1 (Bottom; NC_010392). Regions of nucleotide similarity (BLASTN) on the same strand are highlighted in gray while regions of similarity on the opposite strand are highlighted in turquoise. BLASTN percentage identity is scaled according to the gradient bar. Coding sequences are color-coded according to the figure key. The sequences encoding *gogB* and *sarA* are annotated in the figure. Scale bar indicates genome length. Image was generated using Easyfig (19).

Alignment of a representative of the Gifsy-1-like (GF-1L) prophage sequence to Gifsy-1 (NC_010392.1) showed 98% nucleotide identity over 55% query coverage, with regions of similarity localized to the genes required for lysogeny, DNA-packaging, head morphogenesis, and tail morphogenesis (Fig. 2). The GF-1L prophage was inserted in the 5′ end of the *lepA* gene, previously noted as the insertion site for Gifsy-1 in STM (14). Significantly, the GF-1L prophage was carried by all 197 isolates of SalEnt-18-0030, as well as three isolates without an assigned core SNP cluster. Of the isolates carrying GF-1L that were not assigned a core SNP cluster, all were closest by core SNP distance to SalEnt-18-0030. Alignment of the 200 assembled GF-1L sequences showed high nucleotide similarity, with nine sequences containing ≤7 SNPs relative to the most prevalent sequence shared by the remaining 191 isolates. All of the GF-1L prophages in the outbreak cluster were found to be carrying the prophage associated virulence factor *gogB* (15) but not the anti-inflammatory mediator gene *sarA* (16) (Fig. 2). A previous study in Queensland (QLD), Australia, highlighted a clade of S. Enteritidis that also harbored a Gifsy-1-like prophage (17). Genomic comparisons between our representative GF-1L prophage and the most similar phage (ERR2118167, isolated in 2017) from QLD showed >98% nucleotide identity over 45% of the length of GF-1L. As such, both prophages resemble Gifsy-1, but are genetically distinct, and the QLD prophage did not harbor *gogB* (data not shown).

### Conclusions.

Routine genomic surveillance of S. Enteritidis in NSW, Australia, captured a significant community outbreak with unique features of the accessory partition of the pangenome. The pangenome graph indicated a significant proportion of the shell pangenome consisted of prophage content. Most significantly, the presence of a Gifsy-1-like prophage (GF-1L) highlighted by the pangenome graph was strongly associated with membership in the outbreak-associated core SNP cluster. The GF-1L prophage found in the outbreak contained the virulence factor, *gogB*, which is commonly found in STM. The GogB effector, secreted by the Type III Secretion System, contributes to intracellular survival of the bacterium through anti-inflammatory effects (15, 18), which enhance the ability of Salmonella to colonize the human intestine. The observation of a ubiquitous *gogB* carried by the GF-1L prophage in an S. Enteritidis outbreak suggests that *gogB*-mediated virulence may have contributed to emergence of the outbreak; however, further investigation would be required to establish whether the GogB effector confers the same survival advantage in S. Enteritidis as has been shown for STM (15). Another anti-inflammatory mediator gene, *sarA*, was recently described alongside *gogB* in a Gifsy-1 prophage in STM (16), suggesting that there may be an evolutionary drive toward anti-inflammatory effectors. From a public health perspective, the results of this study raises the possibility that anti-inflammatory mediated virulence, typically observed in STM, may also be conferred on S. Enteritidis by prophages, and further, that the S. Enteritidis pangenome should be monitored for the appearance of virulence factors mobilized by mobile genetic elements such as *sarA*. Overall, these findings indicate that monitoring of the accessory genome of S. Enteritidis during an outbreak can assist the public health response by revealing the presence of virulence, survival, and other genetic factors that may influence the progression of the outbreak, and the success or failure of possible interventions. Monitoring the accessory partition of the pangenome may also serve as another source of information for clustering of isolates alongside core-based phylogeny.

### Data availability.

Genomes for all 897 isolates have been deposited in SRA (BioProjects PRJNA489746 and PRJNA596817). Accession numbers and associated metadata are included in the supplemental material. Genome assemblies are available for representative sequences containing (i) the GF-1L prophage (SRA accession SRX4652660; GenBank assembly accession GCA_025770365.1, GF-1L is located on contig JAOVTH010000009.1 at positions 16511:67966) and (ii) the ST64B-like prophage (SRA accession SRX17826421; GenBank assembly accession GCA_013251545.1, ST64B-like is located on contig JABQPS010000029.1 at positions 38408:79034).
