# Supplementary material for: Pangenome Analysis of a Salmonella Enteritidis Population Links a Major Outbreak to a Gifsy-1-Like Prophage Containing Anti-Inflammatory Gene gogB
Source: Microbiol Spectr. 2023 Mar 14;11(2):e02791-22. doi: 10.1128/spectrum.02791-22 (PMC10100743; doi:10.1128/spectrum.02791-22)
Supplement: Supplemental file 1 — Supplemental material. Download spectrum.02791-22-s0001.pdf, PDF file, 0.1 MB [file spectrum.02791-22-s0001.pdf]

Pangenome analysis of a *Salmonella* Enteritidis population links a major outbreak to a Gifsy-1-like prophage containing anti-inflammatory effector GogB

Supplementary material

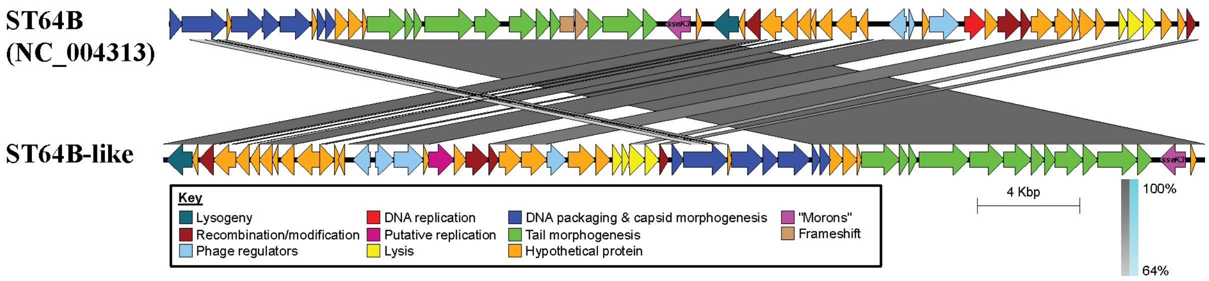

Supplementary Figure 1. Pairwise comparison between the reference phage ST64B (Top; NC_004313) and a representative ST64B-like prophage (Bottom). Regions of nucleotide similarity (BLASTN) on the same strand are highlighted in grey while regions of similarity on the opposite strand are highlighted in turquoise. BLASTN percentage identity is scaled according to the gradient bar. Coding sequences are colour-coded according to the figure key. The coding sequence encoding the SseK3 effector protein is annotated in the figure. Scale bar indicates genome length. Image was generated using Easyfig (17).

Supplementary Table 1. Metadata associated with isolates included in the analysis. Date of specimen collection, membership in the outbreak core SNP cluster, and *in silico* detection of GF-1L and the ST64B-like prophage are indicated. Reads for all isolates are deposited in SRA. Isolates in BioProject PRJNA596817 additionally have assembled genomes deposited.

Supplementary Table 2. Isolates for which the assembled genome are associated with BioProject PRJNA596817 while the reads are instead associated with BioProject PRJNA489746. In each case the two listed BioSample accession numbers refer to the same specimen and library.
